# Extracorporeal membrane oxygenation (ECMO) in the management of non-surgical patients with cardiogenic shock and cardiac arrest

**DOI:** 10.1186/2197-425X-3-S1-A845

**Published:** 2015-10-01

**Authors:** P Ostadal, A Kruger, D Vondrakova, M Janotka, P Kmonicek, M Mates, M Skabradova, P Jehlicka, D Doubek, P Neuzil

**Affiliations:** Cardiovascular Center, Na Homolce Hospital, Prague, Czech Republic; Na Homolce Hospital, Prague, Czech Republic

## Introduction

Veno-arterial extracorporeal membrane oxygenation (VA ECMO) has been introduced in the management of critical conditions caused by severe cardiac failure. However, literary evidence for the use of VA ECMO in non-surgical patients remains insufficient.

## Objectives

To analyze a group of non-surgical patients with cardiogenic shock and refractory cardiac arrest treated with VA ECMO in cardiovascular center.

## Methods

Between January 2006 and March 2015 one hundred and thirty-eight patients were treated in our institution with mini-invasive active circulatory support or VA ECMO for circulatory failure. We analyzed data from a subgroup of 87 primarily non-surgical patients (mean age 61 (31-84) years, 80% were males), treated by VA ECMO.

## Results

The major indication for circulatory support therapy was cardiogenic shock, followed by refractory cardiac arrest, arrhythmic storm, and support of high-risk interventions. Median duration of circulatory support was 3 days, maximum 62 days. The all-cause 30-day mortality in our group was 34.5%; in the subgroup of patients with severe refractory cardiogenic shock the 30-day mortality was 49.4%. In patients with refractory cardiac arrest, where ECMO was introduced during continuous chest compressions, 4 individuals from 18 treated survived with good neurological outcome. We found significant survival differences between subgroup with urgent circulatory support introduction and patients with semi-urgent support (30-day mortality 44.1% vs. 5.3%, P < 0.001). We observed significantly higher lactate levels prior to ECMO insertion in survivors in comparison with non-survivors (P < 0.05); other baseline characteristics including age or left-ventricle ejection fraction were comparable. We evaluated also the role of cerebral/peripheral near-infrared spectroscopy (NIRS) oximetry in the non-invasive monitoring of global circulatory status in patients with mini-invasive circulatory support.

## Conclusions

VA ECMO is a promising tool in the management of severely compromised patients with rapidly progressing cardiogenic shock or refractory cardiac arrest. Frequently the circulatory support therapy in these high-risk patients represents the last chance to survive.

## Grant Acknowledgment

This study was supported by the grant from the Czech Ministry of Health, Nr. 12153 and by the Institutional grant MH CZ - DRO (Nemocnice Na Homolce - NNH, 00023884)

